# Comprehensive assessment of left ventricular myocardial function by two-dimensional speckle-tracking echocardiography

**DOI:** 10.1186/s12947-018-0135-x

**Published:** 2018-09-18

**Authors:** Vicente Mora, Ildefonso Roldán, Elena Romero, Diana Romero, Javier Bertolín, Natalia Ugalde, Carmen Pérez-Olivares, Melisa Rodriguez-Israel, Jana Pérez-Gozalbo, Jorge A. Lowenstein

**Affiliations:** 1 Cardiology Department, Hospital Dr Peset, Valencia, Spain; 2 Cardiodiagnosis Department Medical Research of Buenos Aires, Buenos Aires, Argentina

**Keywords:** Deformation imaging, Speckle tracking, Ventricular torsion, Ventricular function, Myocardial strain

## Abstract

**Background:**

Left ventricular ejection fraction (LVEF) results from the combined action of longitudinal and circumferential contraction, radial thickening, and basal and apical rotation. The study of these parameters together may lead to an accurate assessment of the cardiac function.

**Methods:**

Ninety healthy volunteers, categorized by gender and age (≤ 55 and >  55 years), were evaluated using two-dimensional speckle tracking echocardiography. Transversal views of the left ventricle (LV) were obtained to calculate circumferential strain and left ventricular twist, while three apical views were obtained to determine longitudinal strain (LS) and mitral annular plane systolic excursion (MAPSE). We established the integral myocardial function of the LV according to: 1. The Combined Deformation Parameter (CDP), which includes Deformation Product (DP) - Twist x LS (° x %) - and Deformation Index (DefI) -Twist / LS (° / %)-; and 2. the Torsion Index (TorI): Twist / MAPSE (° / cm).

**Results:**

The mean age of our patients was 50.3 ± 11.1 years. CDP did not vary with gender or age. The average DP was − 432 ± 172 ° x %, and the average DefI was − 0.96 ± 0.36 ° / %. DP provides information about myocardial function (normal, pseudonormal, depressed), and the DefI quotient indicates which component (s) is/are affected in cases of abnormality. TorI was higher in volunteers over 55 years (16.5 ± 15.2 vs 13.1 ± 5.0 °/cm, *p* = 0.003), but did not vary with gender.

**Conclusions:**

The proposed parameters integrate values of twisting and longitudinal shortening. They allow a complete physiological assessment of cardiac systolic function, and could be used for the early detection and characterization of its alteration.

## Background

Assessment of LV systolic function is the cornerstone of an echocardiographic examination. Many parameters can be used for clinical and research purposes, but LVEF is the most commonly employed. LVEF results from the combined action of longitudinal and circumferential contraction, radial thickening, and basal and apical rotation. However, LVEF has many limitations as a parameter, some of them related to imaging techniques and others to the definition itself [1, 2].

The particular double helical structure of myocardial fibers results in systolic rotation of the base and apex of the LV in opposite directions round its longitudinal axis, and the algebraic subtraction of this rotation causes twisting of the heart muscle. Due to this muscular movement, the base of the LV moves towards the apex, producing a longitudinal shortening of the LV [3]. Therefore, systolic ventricular contraction results from the combined and simultaneous action of twisting and shortening of the ventricle. Myocardial torsion - the consequence of these movements - is a fundamental component of cardiac function [4–6].

Speckle tracking echocardiography [7] (STE) is a useful echocardiographic tool for evaluating myocardial function, due its high temporal and spatial resolution, good inter and intraobserver reproducibility [8], the advantage of being independent of the insonation angle [9] and the fact that it is not affected by translational movements of the heart. Furthermore, due to its three-dimensional modality, it allows simultaneous assessment of the entire myocardium of the left ventricle [10].

Initially, rotational mechanics of the LV were measured using invasive approaches [11, 12]. However, during the last two decades, non-invasive imaging techniques have become available for this purpose, with cardiac magnetic resonance constituting the gold standard [13, 14]. The incorporation of STE into clinical practice has rekindled study of the LV’s rotational mechanics [15–17], and a very good correlation with cardiac resonance has been reported (*r* = 0.93) [15, 16].

It has been suggested that, in the future, a combined approach in which both changes in LV rotational mechanics and longitudinal shortening are evaluated and interpreted in an integrated way will be necessary to establish normal reference values [18–20]. The calculation of ventricular torsion from rotation and longitudinal strain by means of STE can provide complementary information about systolic ventricular function in relation to the traditional parameters used in daily practice, such as LVEF [6].

We present values of strain and rotational mechanics obtained with STE in a healthy population. Based on these values, new parameters are proposed for the study of cardiac function, implying a more integral evaluation of the components involved in the mechanism of contraction.

## Methods

### Study population

This is an observational, prospective study in which 104 healthy volunteers were initially enrolled after being randomly selected among hospital staff. Inclusion criteria were: age > 18 years, absence of cardiovascular disease and/or risk factor, and normal physical and electrocardiographic examinations. Exclusion criteria were sports training and pregnancy.

The hospital’s Clinical Research and Ethics Committee approved the study. The written informed consent of the participants was obtained.

### Echocardiography

Two ultrasound systems (Vivid E9 and Vivid E95, GE Healthcare Medical Systems, Norway) equipped with 2.5 MHz transducer were used. Two-dimensional views of the apex (four, three and two chambers) were obtained to calculate longitudinal strain (LS). From the parasternal short axis perspective, transverse projections of the mitral valve, papillary muscles and apex facilitated calculation of radial strain, circumferential strain and rotational parameters. All images were obtained at a frequency of between 50 and 80 frames/second. The moment of aortic valvular closure was determined in the long axis apical projection. All images were transferred to a workstation for computer analysis (EchoPAC GE Healthcare software version 112.0.0).

The LV endocardial border was traced manually slightly within the myocardium to calculate myocardial strain. Then, with the help of the software, a second larger concentric circle was automatically generated near the epicardium to include the full thickness of the myocardial wall. The program automatically divided each projection into six equal segments and performed frame-by-frame speckle tracking, providing automated tracking confirmation (verified by the operator) and generating strain values, expressed as percentages.

Rotation is an angular displacement of a myocardial segment in a transverse projection around the longitudinal axis of the LV. Apical systolic rotation occurs in a counter-clockwise direction and is expressed in degrees with positive values when viewed from the vertex. Conversely, basal rotation is produced in a clockwise direction, with negative values. The twist that occurs as a consequence is the net difference, in degrees, between apical and basal rotation.

Care was taken to ensure that all ultrasound adjustments, including image depth and frame rate, were maintained for each individual’s data collection. Images of four and two chambers were recorded for analysis of end-diastolic and end-systolic volumes, and LVEF was calculated using Simpson’s biplane method.

In addition to LS, longitudinal shortening of the LV was estimated from the systolic excursion of the mitral annular plane (MAPSE) in the four-chamber projection by placing the M-mode cursor at the septal and lateral level of the ring and averaging both values. The base-apex distance (B-A) was determined in the four-chamber view, at the end-diastole and at the beginning of the QRS.

**Fig. 1:**
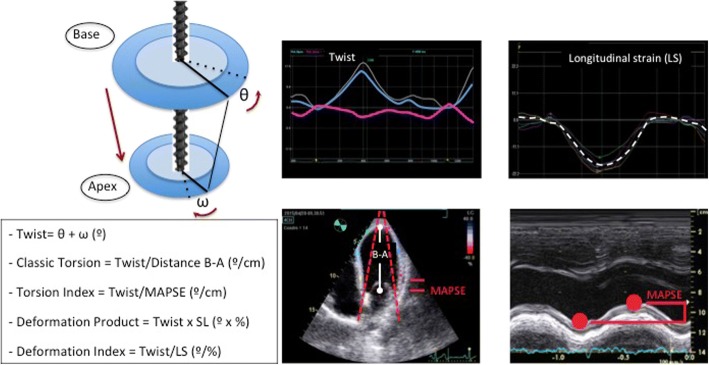
Graphic representation of the LV. Parameters that contribute to LV myocardial function. Abbreviations in text

We evaluated the various parameters that contribute to LV myocardial function (Fig. 1):

- Classic Torsion (CTor): Twist/B-A distance (degrees/cm)
- Torsion Index (TorI): Twist/MAPSE (degrees/cm)
- Combined Deformation Parameter (CDP):
  1. Deformation Product (DP): Twist x LS (degrees x %), and
  2. Deformation Index (DefI): Twist / LS (degrees / %).

### Statistical analysis

Continuous variables are expressed as mean and standard deviation, and proportions as percentages. We compared strain values according to gender and age (≤ 55 years and >  55 years) using a Student’s t test. Normality of distribution of the studied parameters was confirmed. A *p* value of < 0.05 was considered statistically significant. The intraclass correlation coefficient was used to evaluate intraobserver and interobserver reproducibility of continuous variables, according to a random sample of 10 cases, with masking and measurements performed at different moments.

Statistical analyses were performed using the IBM SPSS Statistics v.19.0.0329 software package.

## Results

Our study population was eventually composed of 90 healthy subjects after 14 of the original 104 were excluded due to a deficient ultrasonic window that made it impossible to obtain adequate measurements in all segments of the LV. Mean age was 50.3 ± 11.1 years, women 43.3%.

Table 1 details the population’s characteristics. Women showed lower values for body surface, LV diameter and ventricular volume, while no gender differences were observed in terms of ejection fraction. Patients over 55 years of age presented higher LVEF, despite having smaller ventricular diameters and volumes.

**Table 1:** Characteristics of the study population, differentiated by sex and age

	Total (n 90)	Men (n 52)	Women (n 38)	*p*	≤ 55 years (n 59)	> 55 years (n 31)	*p*
BS (m^2^)	1.8 ± 0.2	1.9 ± 0.1	1.6 ± 0.1	0.01	1.8 ± 0.2	1.8 ± 0.1	0.42
HR (b/m)	65 ± 10	65 ± 11	66 ± 9	0.65	65 ± 11	67 ± 10	0.32
sBP (mmHg)	119 ± 16	121 ± 16	115 ± 15	0.07	117 ± 17	123 ± 15	0.11
LVd (mm)	45.5 ± 4.5	47.4 ± 4.2	43.0 ± 3.4	0.01	46.1 ± 4.5	44.3 ± 4.1	0.06
LVEDV (ml)	89.2 ± 28.4	101.1 ± 27.9	73.0 ± 20.1	0.01	96.1 ± 29.3	76.2 ± 21.7	0.01
LVESV (ml)	29.8 ± 11.1	34.1 ± 11.5	24.1 ± 7.5	0.01	32.9 ± 11.4	24.1 ± 8.1	0.01
LVEF (%)	66.6 ± 5.5	66.4 ± 5.4	66.9 ± 5.7	0.68	65.7 ± 5.3	68.4 ± 5.7	0.03

*BS* Body surface area, *HR* Heart rate, *sBP* systolic blood pressure, *LVd* Left ventricle end-diastolic diameter, *LVEDV* Left ventricular end-diastolic volume, *LVESV* Left ventricular end-systolic volume, *LVEF* Left ventricular ejection fraction

The mean values of LS, radial strain and circumferential strain are presented in Table 2. LS was the only of the 3 types of strain that was slightly higher in the female sex, while none showed differences according to age. MAPSE was higher among males and in the ≤55 year-old group. Neither the values of basal and apical rotation, nor the resulting twist, differed with gender or ages.

**Table 2:** Values of global longitudinal, radial and circumferential strain, and rotational parameters of the study population, differentiated by sex and age

	Total (n 90)	Men (n 52)	Women (n 38)	*p*	≤ 55 y (n 59)	> 55 y (n 31)	*p*
LS (%)	−21.1 ± 2.1	− 20.7 ± 2.0	−21.7 ± 2.1	0.02	−21.1 ± 1.8	−21.1 ± 2.5	0.98
RS (%)	33.5 ± 10.2	34.0 ± 9.9	32.8 ± 10.7	0.59	32.4 ± 9.6	35.6 ± 11.2	0.15
CS (%)	−21.6 ± 3.9	−21.9 ± 4.3	−21.3 ± 3.4	0.41	−21.2 ± 3.4	− 22.4 ± 4.7	0.16
MAPSE (cm)	1.4 ± 0.1	1.48 ± 0,1	1.36 ± 0.1	0.01	1.4 ± 0.2	1.3 ± 0.1	0.01
B-A (cm)	8.2 ± 0.8	8.6 ± 0.6	7.6 ± 0.6	0.01	8.3 ± 0.8	7.9 ± 0.8	0.04
Apical Rot (°)	14.4 ± 6.5	14.9 ± 7.0	13.7 ± 5.9	0.40	14.2 ± 1.0	14.7 ± 0.9	0.76
Basal Rot (°)	− 6.2 ± 3.6	−6.0 ± 3.5	−6.5 ± 3.8	0.50	−5.7 ± 3.4	− 7.3 ± 3.9	0.05
Twist (°)	20.3 ± 7.6	20.7 ± 7.9	19.6 ± 7.1	0.51	19.4 ± 7.9	21.9 ± 6.6	0.14
CTor (°/cm)	2.5 ± 1.0	2.4 ± 1.0	2.6 ± 1.0	0.39	2.3 ± 1.0	2.8 ± 0.9	0.05
TorI (°/cm)	14.2 ± 5.3	14.0 ± 5.4	14.5 ± 5.2	0.67	13.1 ± 5.0	16.5 ± 15.2	0.01
CDP							
DP (° x %)	−432 ± 172	− 431 ± 170	433 ± 177	0.96	− 415 ± 182	− 463 ± 149	0.21
DefI (° / %)	− 0.96 ± 0.36	−1.00 ± 0.39	−0.90 ± 0.31	0.18	−0.9 ± 0.3	−1.0 ± 0.3	0.09

*LS* Longitudinal Strain, *RS* Radial Strain, *CS* Circumferential Strain, *MAPSE* Mitral annular plane systolic excursion, *B-A* Base-Apex distance, *Rot*. Rotation, *CTor* Classic Torsion, *TorI* Torsion Index, *CDP* Combinated Deformation Parameter, *DP* Deformation Product, *DefI* Deformation Index

### Behaviour of torsional parameters

CTor, TorI and DefI did not vary with sex, while age did have a bearing: CTor and TorI values were higher in older subjects (2.8 ± 0.9 vs 2.3 ± 1.0, *p* = 0.05, and 16.5 ± 15.2 vs 13.1 ± 5.0, *p* = 0.003, respectively), while no age differences were observed with respect to DefI (Table 2).

CDP showed no gender or age differences in either DP or DefI. The possible combinations between DP and Defl values appear in Table 3. Normal values of DP (− 432 ± 172 ° x %) and DefI (− 0.96 ± 0.36 °/%) relate to a cardiac function within normal limits. When there is myocardial dysfunction, the DP value can be pseudonormal or decreased, and so DefI is a better parameter of the origin in the involvement of LS, twisting or both. The DP is pseudormal when its value is maintained at the expense of a compensating increase of the twist, with LS diminished. When the DP is decreased, the DefI quotient will provide information on the origin of myocardial impairment in one (LS or twist) or both components.

**Table 3:** Combinated Deformation Parameter. Possibilities of presentation

Combinated deformation parameter			
Deformation Product (DP) (Twist x LS) (° x %)	Deformation Index (DefI) (Twist/LS) (° / %)	Status of Twist and LS	Myocardial function
Normal	Normal	Normal Twist + Normal LS	Normal
Pseudonormal	Increased	Increased Twist + LS diminished	Impaired
Diminished	Diminished	Diminished Twist + Normal LS	Impaired
	Increased	Normal Twist + LS diminished	Impaired
	Normal	Diminished Twist + LS diminished	Impaired

*LS* Longitudinal strain

Intraobserver and interobserver variability was suitable (Table 4), with intraclass correlation coefficients > 0.75.

**Table 4:** Intraobserver and interobserver variability

Intraclass correlation coefficient (95% CI)		
		*p*
Intraobserver		
Global longitudinal strain	0.86 (0.53–0,96)	< 0.001
Twist	0.94 (0.65–0.98)	< 0.001
Classic Torsion	0.96 (0.84–0.99)	< 0.001
Torsion index	0.93 (0.63–0.98)	< 0.001
Strain product	0.97 (0.82–0.99)	< 0.001
Strain index	0.85 (0.52–0.96)	< 0.001
Interobserver		
Global longitudinal strain	0.87 (0.58–0.96)	< 0.001
Twist	0.85 (0.53–0.96)	< 0.001
Classic Torsion	0.87 (0.59–0.96)	< 0.001
Torsion index	0.80 (0.41–0.94)	< 0.001
Strain product	0.90 (0.65–0.97)	< 0.001
Strain index	0.77 (0.31–0.94)	0.003

## Discussion

We describe values of new echocardiographic parameters that can be employed to perform a comprehensive evaluation of cardiac mechanics using a non-invasive technique - STE - in a sample of healthy subjects. In this way we intend to provide a full picture of the physiological components involved in ventricular contraction, and to determine which can serve as tools in the evaluation of different heart diseases.

STE is a useful technique to evaluate cardiac function [7], allowing each myocardial region to be visualized as a defined, relatively stable and unique speckle pattern, thus allowing it to be differentiated from other regions throughout the cardiac cycle. Nevertheless, calculating strain by means of this technique has its limitations, such as the dependence on frame speed for an adequate tracking of the marks [21], and on the quality of the two-dimensional image, as well as myocardium thickness heterogeneity, LV geometry, variations in the resolution of the lateral wall and suboptimal reproducibility [22].

The values obtained with STE are related specifically with LV myocardial function, and allow the mechanism of contraction and its components to be described, such as: longitudinal and circumferential shortening, radial thickening, and rotational movements [2].

The arrival of STE has revived interest in LV torsion, and research shows a very close correlation with cardiac resonance (*r* = 0.93) in humans [15, 16]. Cardiac torsion is a result of the peculiar architecture of the heart; several studies have highlighted the presence of obliquely oriented muscle fibres in the subendocardium, which gradually change their angle so that they position themselves in the opposite direction to the subepicardium [3, 23, 24]. This spiral organization seems to be fundamental for both cardiac and diastolic systolic function [25, 26].

In the 1960s, Torrent-Guasp described myocardial architecture as a single band of muscle that forms a double helix [27], allowing simultaneous systolic rotation in opposite directions between the LV base and apex, around its longitudinal axis. At the same time, the ventricular base moves towards the apex, which induces a longitudinal shortening of the LV and, consequently, ventricular torsion [3, 28]. Therefore, systolic ventricular contraction results from the combined and simultaneous action of ventricular rotation and longitudinal shortening (Fig. 1). The two processes may not be affected in the same way by ventricular dysfunction [29], so their evaluation may represent a valuable tool for clinical practice, even in moderate pathological situations that are not detected by analysing classical hemodynamic parameters [17, 26, 30–32].

There is some semantic confusion in the literature surrounding the indistinct usage of the terms twist and torsion. Twist is the result of the algebraic difference between basal and apical rotation, and is expressed in degrees, while classic torsion (CTor) is usually defined by dividing the twist by the B-A distance during the final phase of the diastole, expressed in degrees/cm [33]. According to another definition, this formulation should be multiplied by the average radius of the base and the apex [34] to adjust for hearts of different longitudinal and transverse size. In both cases, the twist is normalized with respect to one or several “static” LV diameters, so that one of the actions that take place simultaneously during the systole - namely, the shortening of the LV along its longitudinal axis - is not taken into account. However, it is possible to evaluate the “dynamic” longitudinal shortening of LV by calculating the MAPSE using conventional 2D echocardiography, and by estimating LS by means of EST.

The parameters proposed in this work for the evaluation of ventricular torsion, such as TorI and DefI, provide a more reliable representation of the components of cardiac systole. They involve quantification of longitudinal shortening and twist, as an expression of dynamic systolic events that occur simultaneously in an elastic organ such as the ventricular myocardium.

It is important to underline the difference between the meanings of the commonly used definition classic torsion (CTor) [34] and the proposed indexes of torsion (TorI and DefI). CTor refers to the twist per cm of the length of (B-A) of the LV, while TorI and DefI represent the rotation per unit of shortening and unit of longitudinal deformation of the LV, respectively. Basal and apical rotations, which produce the twist, are determined both by the segmental heterogeneity of the circumferential contraction and by its interaction with the simultaneous longitudinal contraction, which displaces the circumferential plane obliquely towards the apex during systole. The resulting LV torsion is the outcome of the longitudinal-circumferential shear.

The torsion generated as a result of this interaction, and which depends on the helical shape of the myocardial fibers, contributes to the thickening of the myocardium towards the ventricular centroid and the consequent ejection of the corresponding systolic volume. Therefore, with the proposed indices (TorI and DefI), we intend to provide a measure of intrinsic muscular torsion that is independent of cardiac size. CTor normalizes twist with respect to a static dimension such as the diastolic size of the LV. Thus, the extent of the torsion may be reduced according to its classical definition (with respect to the longitudinal size of the LV) (CTor) and increased when calculated by TorI and DefI, as can be appreciated in Table 5. This table shows the evolution of a patient with cardiac amyloidosis: a decrease in the twist, when accompanied, as it usually is, by a decrease in ventricular longitudinal shortening, can actually lead to an increase in intrinsic myocardial torsion known as “writhing”, similar to the “squeezing” of a towel. In patients diagnosed with amyloidosis, an increase of TorI and DefI (greater “squeezing”) justifies maintenance of the LVEF, despite the decreased twist and reduced contraction of the longitudinal axis. Hence, the indices we propose are more representative when defining the alterations of the ventricular torsion referred to as “squeezing”, since they contemplate dynamic simultaneous actions like longitudinal shortening and twist.

**Table 5:** Evolution in a case of amyloidosis. Normal values of reference in Table 2

	Twist (°)	LS (%)	B-A (cm)	MAPSE (cm)	CTor (°/cm)	TorI (°/cm)	Combined deformation parameter		LVEF (%)
							DP (° x %)	DefI (° / %)	
Basal	20	−14	8	0.9	2.5 (N)	22.2 (↑)	− 280 (↓)	− 1.4 (↑)	55%
1 year	15	−10	8	0.7	1.8 (↓)	21.4 (↑)	− 150 (↓)	− 1.5 (↑)	55%
2 years	8	−8	8	0.6	1.0 (↓)	13.3 (N)	−64 (↓)	−1 (N)	48%
3 years	5	−7	8	0.5	0.6 (↓)	10.0 (↓)	−35 (↓)	−0.7 ↓	35%

From the beginning and throughout follow-up a low DP is an indicator of myocardial dysfunction, with normal LVEF. In the baseline diagnosis, a greater TorI and DefI are observed (with respect to the normal values in Table 2), reflecting an increase in myocardial torsion, which compensates to maintain the LVEF. This is not detected by the CTor measure, which appears normal. As the disease progresses, the normalization and subsequent diminishing of TorI and DefI are accompanied by lower LVEF, which reflects the exhaustion of the compensating mechanism. CTor shows less sensitivity in the detection of systolic dysfunction during follow-up

*LS* Longitudinal Strain, *B-A* Base-Apex distance, *MAPSE* Mitral annular plane systolic excursion, *T* Twist, *CTor* Classic Torsion, *TorI* Torsion Index, *DP* Deformation Product, *DefI* Deformation Index, *LVEF* Left Ventricular Ejection Fraction, *N* normal

↑: Increased, ↓: Diminished

However, MAPSE is not always feasible; namely, in patients with mitral prosthesis or calcification of the mitral annulus. As a result of this limitation, together with the fact that the LS and twist can be visualized with the same speckle tracking technology base, it is preferable to use DefI. In addition, MAPSE and LS do not always occur in parallel, and the latter has already demonstrated itself to be easily measured and to have prognostic capacity when considered in isolation [35–38].

We propose the “Combined Deformation Parameter” (CDP) for the estimation of myocardial function throughout the evaluation of the two fundamental components of contractile mechanics: twist and LS. The CDP consists of two aspects: first of all, the state of myocardial function, evaluated as the “Deformation Product” (Twist x LS), which quantifies the longitudinal deformation and the twist, both of which increase contractility. Secondly, the extent of the involvement of each component in ventricular function is assessed by the DefI quotient (Twist/LS). The different possible combinations are shown in Table 3. The “Deformation Product” may be normal, pseudormal (when its value is maintained at the expense of a compensating increase of the twist), or decreased. When the DP is pseudonormal or decreased, the DefI quotient will provide information on the origin of myocardial impairment in one or both components. These values may be altered before ventricular dysfunction occurs, and so may be of relevance for the early diagnosis and monitoring of some heart diseases.

The CDP may be useful for monitoring cardiopathies characterized by ventricular volume overload, in which LS may not represent the earliest alteration nor be the only alteration. Similarly, just as the study of cardiotoxicity in oncology is mainly based on the evaluation of EF and LS, the CDP may be an asset in the evaluation of transmural myocardial conditions that are not limited to LS.

In this sense, heart failure has also recently been classified according to alterations in the mechanical function of the LV [39]. After having observed anomalous specific patterns of ventricular myocardial mechanics in different subsets of patients with heart failure, an alternative approach has been proposed for its characterization [39, 40]. In this way, heart failure can be classified into three large subgroups: 1. Predominant longitudinal dysfunction; 2. Transmural dysfunction (longitudinal and circumferential); and 3. Predominant circumferential dysfunction. This classification is based on the orientation of the myocardial fibers of the LV, which are arranged obliquely in a double helix shape. Endocardial fibers, which are aligned in a more parallel fashion to the LV long axis, are mainly associated with longitudinal mechanics, while transmural fibers are mainly responsible for circumferential mechanics [41]. The action of the latter is predominant due to its greater radius of action.

Cardiac structural and functional changes during the early stages of heart failure can act as compensatory mechanisms. However, as the disease progresses, these mechanisms are often insufficient. When evaluated in isolation, LS has proven its prognostic capacity [42, 43] as the most important echocardiographic predictor of cardiovascular death and/or heart failure in the PARAMOUNT and TOPCAT trials of patients with heart failure and preserved EF [38, 44]. LVEF and global circumferential strain, the main determinant of ventricular twist, were preserved in the patient population, suggesting it acted as a compensatory mechanism. The echocardiographic characterization of these changes provides a framework for key management decisions [19, 45]. CDP could be useful in this respect due to their capacity to identify and quantify the normality or abnormality of myocardial function and to determine to what extent the different components of the heart are affected.

There is a consensus that future practice needs to adopt a combined approach in which changes in LV rotational mechanics and longitudinal shortening are considered and interpreted together, and normal values will need to be established if this to be possible [10, 19]. We show LV torsion to be a reliable integral measure of the early qualitative and quantitative detection of cardiac dysfunction. A standardized method of calculating LV torsion that is capable of providing reproducible and comparable measurements needs to be adopted before it can be used as a clinical tool for the diagnosis of myocardial dysfunction [17]. Likewise, the integration of complementary parameters of pumping and myocardial function should be considered for a more accurate evaluation of LV systolic function.

## Conclusions

The proposed parameters integrate values of twisting and longitudinal shortening. In the present work, we provide reference values obtained in a population of healthy subjects. These parameters allow a complete physiological assessment of cardiac systolic function, and could be used for the early detection and characterization of its alteration.

### Limitations

Despite the great advantages offered by 2D-STE, it does have several limitations. It depends on the quality of the image, produces inaccuracies due to planar movement, the quality of the tracking is usually lower at the distal level compared to nearby fields, and frames that are too high or too low are associated with poor tracking. Some limitations are overcome with 3D-STE [46], but a lower temporal resolution, greater susceptibility to image quality in the grey scale and lack of experience are among the remaining challenges.

The sample size is limited so that the new parameters deserve more extensive validation. Maybe a future, multicentre study could provide a definite answer regarding the usefulness of the proposed parameters. Likewise, the value of the new parameters should be validated against other methodologies, such as magnetic resonance imaging.

## Abbreviations

B-A: Base-apex distance
CDP: Combined deformation parameter
CTor: Classic torsion
DefI: Deformation index
DP: Deformation product
LS: Longitudinal strain
LVEF: Left ventricular ejection fraction
MAPSE: Mitral annular plane systolic excursion
STE: Speckle tracking echocardiography
TorI: Torsion index
